# Unveiling FLNC variants: iPSC-derived myogenic cells as a model to study disease mechanisms

**DOI:** 10.1186/s13395-026-00418-5

**Published:** 2026-02-12

**Authors:** Nassam M. Daya, Anne Schänzer, Andreas Hentschel, Marie-Cecile Kienitz, Dominik Sellung, Nicolina Suedkamp, Karsten Krause, Jaqueline C. Kinold, Leon Volke, Anja Schreiner, Hanna Schlierbach, Christopher Nelke, Felix Kleefeld, Anne-Katrin Guettsches, Holm Zaehres, Tobias Ruck, Lampros Mavrommatis, Andreas Roos, Matthias Vorgerd

**Affiliations:** 1 Ruhr University Bochum, BG University Hospital Bergmannsheil, Department of Neurology, Bochum, Germany, Ruhr-University Bochum, Bochum, Germany; 2 BG University Hospital Bergmannsheil, Heimer Institute for Muscle Research, Bochum, Germany, BG University Hospital Bergmannsheil, Bochum, Germany; 3 Institute of Neuropathology, Justus-Liebig-University Giessen, DE, Justus Liebig University, Giessen, Germany; 4 Translational Neuroscience Network Giessen (TNNG), Justus-Liebig-University, Giessen, Germany; 5 Leibniz-Institut Für Analytische Wissenschaften-ISAS E.V., Dortmund, 44139 Germany; 6 Department of Cellular and Translational Physiology, Medical Faculty, Ruhr-University Bochum, Bochum, Germany; 7 Department of Neurology, Medical Faculty, University Hospital Düsseldorf, Heinrich Heine University, Düsseldorf, 40225 Germany; 8 Charité - University Medicine Berlin, Berlin, Germany; 9 Department of Anatomy and Molecular Embryology, Institute of Anatomy, Ruhr-University Bochum, Bochum, 44801 Germany; 10 Department of Pediatric Neurology, Center for Neuromuscular Disorders, University Duisburg- Essen, Essen, 45147 Germany; 11 Brain and Mind Research Institute, Children’s Hospital of Eastern Ontario Research Institute, Ottawa, ON K1H 8L1 Canada

**Keywords:** Disease modeling, Filaminopathies, iPSC, Organoid

## Abstract

**Background:**

Filaminopathies, caused by pathogenic *FLNC* variants, are rare neuromuscular disorders characterized by protein aggregation, z-disk pathology and lead to progressive muscle weakness and/or cardiomyopathies.

**Methods:**

To address the lack of existing filaminopathy models in skeletal muscle, we developed a patient-specific cellular platform using induced pluripotent stem cells (iPSCs) harboring two truncating filamin C (FLNc) variants (p.Q1662X, p.Y2704X). Employing a developmental human skeletal muscle organoid hSMO model, we enrich for myogenic progenitor cells that are further differentiated into functional myotubes through 2D and 3D approaches (myotubes and musculoids).

**Results:**

The 2D myotubes exhibited poor sarcomeric organization and hallmarks of filaminopathies, including protein aggregation and proteostatic dysfunction, marked by elevated aggresome formation and an increased basal autophagic flux. The 3D musculoids revealed ultrastructural abnormalities and enabled the identification of novel disease-associated proteins involved in ER stress and protein folding (e.g. DNAJC10) through proteomic analysis. Proteomic findings were additionally validated in 2D cultures and in corresponding patient-derived muscle biopsies enhancing the model’s translational value.

**Conclusions:**

Our model is suitable to monitor aspects of filaminopathies’ pathogenesis and to investigate possible therapeutic interventions with quantitative readouts.

**Supplementary Information:**

The online version contains supplementary material available at 10.1186/s13395-026-00418-5.

## Introduction

Recent scientific advancements have facilitated the detection and characterization of numerous (rare) diseases including neuromuscular disorders. These analytical approaches have not only led to an expanded understanding of the pathophysiology but have also revealed valuable starting points for potential therapeutic intervention concepts [1]. However, these studies are often complicated by the diverging and complex effects of the underlying genetic variants: different pathogenic variants in the same gene can result in variable protein structures, trigger different pathophysiological cascades and even lead to different clinical manifestations or altered ages of disease manifestation, which in turn reflects on the potential interventions. This necessitates rapid progress in disease modeling of various cell types, enabling future patient-tailored treatments. Skeletal muscle is one of the most abundant tissues in the human body and a wide spectrum of disorders affect its proper function. In vitro studies of skeletal muscle have been for long limited to myogenic cell lines that fuse in culture to form multinucleated tubes [2]. Beside failing to reach a functional maturity level that resembles the human muscle complexity, introducing patient specific mutations into these cell lines can have unintended genetic consequences as it may require significant resources [3]. Reprogramming patient somatic cells into induced pluripotent stem cells (iPSCs) helps overcome numerous challenges that are faced with previously existing myoblast cell lines [4]. Resetting the human developmental potential of adult tissues via generating iPSCs for patient and healthy specific tissues provides a robust cell population amenable to developmental cues for achieving reliable and reproducible protocols for modeling skeletal muscle in vitro. Attempts at recapitulating in vitro skeletal muscle development from iPSCs in two-dimensions by sequential introduction of growth factors and cytokines at specific developmental onsets [5–7] were followed by adaptation of culture condition and developmental cues in a three-dimensional environment by Mavrommatis and colleagues. This three-dimensional organoid approach of skeletal muscle development, resulted in the establishment of committed myogenic populations with presence of a myogenic progenitor/satellite-like cell population expressing Pax7, hereafter referred to as human skeletal muscle organoid (hSMO) [8, 9] and was applied herein to investigate filaminopathies.

Filamin C (FLNc) is a member of the actin binding filamins A, B and C. It is predominantly expressed in skeletal and cardiac muscle tissue. FLNc is mainly localized in z-disk structures of striated muscle and thus plays a crucial structural role. Its presence in costameres further elucidates its functions in linking the contractile units of the muscle cells to the sarcolemma and extracellular matrix, contributing to the structural integrity of muscle cells and facilitating the transmission of mechanical force [10]. Up to date, over 500 different mutations were identified in the *FLNC* gene yielding in different clinical phenotypes [11] including myofibrillar myopathies (MFM) (MIM: 609524). MFM progress slowly and result in skeletal muscle weakness, due to intracellular protein aggregations in striated muscle fibers [12, 13]. Recently, two novel pathogenic *FLNC* variants were identified, (c.4984 C > T ◊ p.Q1662X) and (c.8112delC ◊ p.Y2704X), both causative for a slowly progressive myopathy with an adult onset and accompanied by aggregate formation in histological findings [14]. The aggregate formation is believed to be one major pathophysiological driver impacting on overall proteostasis including the protein quality control system [15]. Thus, variable clearance systems in the cell might be utilized according to the different mutations [14]. In the quest for further understanding of the two recently identified disorders, the hSMO developmental model was applied on patient derived p.Q1662X [16], and p.Y2704X hiPSCs [17]. Enrichment of myogenic-lineage cells was obtained by Fluorescence-activated cell sorting (FACS) after purifying for CD82 positive populations (CD82+), a marker that at that stage delineates skeletal muscle lineage within the hSMO culture [18, 19]. CD82 + cells are further expanded and differentiated in 2D cell culture to form multinucleated myotubes. Unlike traditional in vitro models [2], our myotubes show sarcomeric organization, respond to acetylcholine and exhibit a phenotype resembling filaminopathies. To better recapitulate native muscle architecture, the CD82 + cells were embedded in Matrigel^®^ droplets, allowing them to grow and differentiate in a three-dimensional environment and form organoids termed musculoids. Ultrastructural examination of the musculoids revealed perturbed organization of myofibrils, streaming of z-disks and variation in sarcomeric length in the pathogenic *FLNC* samples. Furthermore, proteomic analysis indicated impaired differentiation in *FLNC* variant musculoids, marked by reduced expression of key cytoskeletal proteins compared to control. Proteomic profiling also identified novel disease-associated proteins involved in ER stress and protein folding (DNAJC10, HYOU1). These findings were validated in muscle biopsies from corresponding patients by means of immunohistochemical staining and western blotting. Despite the lack of biological replica involved in this study, the verifiable data obtained from the propagated muscle cells in corresponding muscle biopsies renders the model suitable for in vitro studies and disease modelling. The CD82 + cells obtained from 3D-differentiaion of iPSCs into myogenic lineage can be used to investigate other neuromuscular disorders, serve as a platform for drug screening and possible therapeutic interventions.

## Methods

### iPSCs cell culture

iPSCs are cultivated on Matrigel^®^- coated dishes (Corning) using StemFlex™(Gibco™) media. The culture is passaged at 70–80% confluency by enzymatic dissociation using TrypLE™ Select Enzym (Gibco™). Upon seeding, the cells are treated with the ROCK-inhibitor (Y-27632) at 10 µM (StemCell Technologies). Media is refreshed daily. The lines used in this work are CB CD34 iPSCs as an unaffected control line, referred to as ‘’CD34’’ throughout this manuscript [20] and the two FLNc diseased lines HIMRi001-A referred to as ‘’p.Q1662X’’ [16] and HIMRi005-A ‘’p.Y2704X’’ throughout the manuscript [17]. Donor information are summarized in Table 1. The use of reprogrammed human iPSC lines for research was performed after ethical approval from the local ethics commission of the Ruhr University Bochum, Medical Faculty (15-5401, August 2015).

**Table 1:** Overview of used iPSCs cell lines

Cell line	Sex	age	Donor Cells	Method of Reprogramming	Mutation	Clinical Symptoms	Serum Creatine Kinase (ref < 146 U/l)
CB CD34 “CD34)	f	N/A	Cord Blood	Lentiviral Transduction	None	N/A	N/A
HIMRi001-A “p.Q1662X”	m	60	Dermal Fibroblasts	Lentiviral Transduction	*FLNC c.4984 C > T; p.Q1662X*	Myalgia, Exercise intolerance, Gait disturbances.	221
HMRi005-A “p.Y2704X”	f	56	Dermal Fibroblasts	Lentiviral Transduction	*FLNC* c.8112delC; p.Y2704X	Proximal muscle weakness of the lower extremities with positive Gower’s sign	325

### Differentiation of the iPSCs into myogenic lineage in an organoid model (hSMO)

Following the approach established by Mavrommatis et al. [9, 18], embryoid bodies (EBs) were formed using the hanging drop method (Polyvinyl Alcohol-Sigma, Y-27632-StemCell Technologies, StemFlex™-Gibco™, TryplE™ Select Enzym- Gibco™). The following day, the EBs were embedded in Matrigel^®^ (Corning) and cultivated in the basal media DMEM: F12 (PAN Biotech) supplemented with L-glutamine and non-essential amino acids (Thermofisher SCIENTIFIC). Further medium supplementation and Cytokines were introduced to the culture at different time points to direct iPSCs towards myogenic lineage as previously described [9]. Namely, ITS-G 100X (Gibco™), CHIR99021 (Sigma-Aldrich), LDN193189 (Sigma-Aldrich), human recombinant basic fibroblast growth factor bFGF (PeproTech), retinoic acid (Sigma-Aldrich), human recombinant Sonic Hedgehog Shh (PeProTech), human recombinant WNT-1 A (PeproTech), human recombinant hepatocyte growth factor HGF (PeproTech), and ITS-X 100X (Gibco™). Detailed media composition is listed in Table 2. At day 50–60, 10–16 organoids of each cell line were then mechanically dissociated and cultivated using the human skeletal muscle cell growth medium (PromoCell^®^). Dissociated cells are cultivated for 2–3 days prior to myogenic lineage enrichment. Cells of the myogenic lineage were enriched by FACS using the CD82 antibody (Biolegend) at the Department of Molecular Immunology at the Ruhr University of Bochum using the Beckman Coulter MoFlo Astrios cell sorter. The sorted populations - referred to as CD82 + cells- were then used for further experimentations (Supplementary Fig. 1).

**Table 2:** Differentiation of iPSCs into myogenic lineage media components

Basal Media:	DMEM:F12, 2µM Glutamine, 1% non-essential amino acids (v/v), 0.2% Penicillin/Streptomyinc (v/v)	
Supplement:	Added to basal medium and changed at the following days (D)	
D0, D1: 1:100 100X ITS-G 3µM CHIR99021 0.5 µM LDN193189	D3: 1:100 100X ITS-G 3µM CHIR99021 0.5 µM LDN193189 10 ng/ml bFGF	D5: 1:100 100X ITS-G 3µM CHIR99021 0.5 µM LDN193189 10 nM Retinoic Acid 5 ng/ml bFGF
D7, D9: 1:100 100X ITS-G 0.5 µM LDN193189 34 ng/ml SHH 20 ng/ml WNT1A	D11, D13, D15: 1:100 100X ITS-G 10 ng/ ml bFGF 10 ng/ml HGF	D17, >D17: 1: 100 100X ITS-X 10 ng/ML HGF

### 2D differentiation of iPSC-Derived myoblasts (CD82^+^ Cells)

2 × 10^5^ CD82 + cells were seeded in each Matrigel^®^-coated 35 mm µ-Dish (ibidi^®^). Upon 100% confluency the human skeletal muscle cell growth medium (PromoCell^®^) was replaced gradually with skeletal muscle differentiation medium (PromoCell^®^) supplemented with 1X ITS-X (Gibco™), 1X N-2 Supplement (Gibco™) and 5µM of the TGF-ß inhibitor SB431542. Two thirds of the supplemented differentiation medium was replaced every other day until tube formation and spontaneous contraction is observed. Media was changed again to growth medium the last 24 h before end experiments. Table 3. provides detailed information about passage number for each of the differentiation experiments.

**Table 3:** Detailed passage number information for variable experimental approaches

Experiment	CD34_CD82+	*p*.Q1662X_CD82+	*p*.Y2704X_CD82+	Day of experiment (fixing/lysis/measurement)
Immunohistochemical staining	P4/2/2/4/2/2	P3/4/1/2/2	P6/4/1	10
Electrophysiological Measurements	P4/2/2	P3/4/2	P6/2	9
Aggresome Assay 1	P4/2/2/4/2/1	P3/4/1/2/1	P6/4/1	11
Aggresome Assay 2	P4/2/2/4/2/2	P3/4/1/2/2	P6/4/2	12
Aggresome Assay 3	P4/2/2/4/2/4	P3/4/1/2/3	P6/4/3	12
Immunoblotting 1	P4/2/2	P3/4/2	P6/2	11
Immunoblotting 2	P4/2/2/4/2/4	P3/4/1/2/3	P6/4/3	11
Immunoblotting 3	P4/2/2/4/2/5	P3/4/1/2/4	P6/4/4	11
Calcium Imaging	P4/2/2/4/2/2/1	P3/4/1/2/2/1	P6/4/2/1	11
Transmission Electron Microscopy	P4/2/1	P3/4/1	P6/1	35
Proteomics 1	P4/2/1	P3/4/1	P6/1	35
Proteomics 2	P4/2/2/4/2/2/1	P3/4/1/2/2/1	P6/4/2/1	38
Proteomics 3	P4/2/2/4/2/2/1	P3/4/1/2/2/1	P6/4/2/1	38

#### Immunohistochemical staining

Myotubes were fixed with − 20 °C methanol for 5 min followed by -20 °C acetone for 30 s. The solvent was discarded, and the culture dishes were air dried. 5 min incubation with TritonX 0.05% was carried out for further permeabilization of fixed cells. A 10% Normal Goat Serum (NGS), 5% Fetal Calf Serum (FCS) PBS solution was used for blocking for 1 h. FLNc_/_a antibody RR90 [21] in 1:30 dilution (in blocking solution) was incubated over night at 4 °C. The next day, the fixed cells were washed thrice with PBS. Anti-IgA alexa fluor 488 was used 1:100 in blocking solution for 1 h at room temperature (RT). Followed by 3 washing steps to further stain the cells with anti α-Actinin for 1 h RT 1:500 (A7732-Sigma-Aldrich). A corresponding secondary antibody is used after thorough washing for 1 h at RT (Alexa Fluor anti-mouse IgG 568 donkey A10037- Invitrogen). The cell preparations were microscopically examined using the Keyence BZ-X810 microscope (program: BZ-X800 Viewer 1.3.0.1, camera: CCD). For validation studies, similar fixing and staining protocols are carried out on 7 μm sections of muscle biopsies of the patients of interest as well as an unaffected control using the following antibodies (SEC62 [22], HYOU1 [23], DNAJC10: 13101-1-proteintech).

#### Electrophysiological measurements

Membrane currents were measured using whole-cell patch clamp. Pipettes fabricated from borosilicate glass were filled with the solution listed below (pipette resistance, 4–6 MΩ). Currents were measured by means of a patch clamp amplifier (Axopatch 200 A, Axon Instruments) connected to a data acquisition system LIH8 + 8 (HEKA, HEKA Elektronik, Germany) for voltage control and data acquisition. Signals were filtered (corner frequency 1 kHz), digitally sampled at 200 Hz and stored on a computer equipped with the software “Patchmaster” (HEKA, Germany) for data recording and analysis. Experiments were performed at ambient temperature (23–26 °C). Myotubes were voltage-clamped at a holding potential of -90 mV. Application of acetylcholine (ACh) was performed by means of a solenoid-operated flow system, permitting a change of solution around an individual myotube with a half time of about 100 ms. For current measurements, myotubes devoid of contact with neighboring cells were selected. The extracellular solution (in mmol/L) was composed as follows: NaCl 122, KCl 5.4, CaCl_2_ 0.5, MgCl_2_ 1.0, HEPES/NaOH 10.0, pH 7.4. The pipette solution (in mmol/L) was composed as follows: K-aspartate 100, KCl 40, NaCl 5.0, MgCl_2_ 2.0, Na_2_ATP 5.0, EGTA 2.0, GTP 0.025, and HEPES/KOH 20.0 (pH 7.4).

#### Aggresome assay

For detection of aggresome formation the PROTEOSTAT^®^ Aggresome Detection kit was utilized (Enzo, ENZ-51035). A positive control was propagated by treatment with 7.5 µM of the proteasome inhibitor MG-132 for 16 hours prior to fixation with paraformaldehyde (PFA). The myotubes were next fixed with 4% PFA for 30 min at RT on an orbital shaker. After thorough washing with PBS, myotubes were permeabilized on ice using 0.5% Triton X-100, 3 mM EDTA, pH 8.0 in PBS for an additional 30 min. After washing, samples were incubated with the kit’s dual reagents according to the manufacturer’s instructions. The Keyence BZ-X810 microscope was used to examine stained aggresome using the BZ-X800 Viewer program (version 1.3.0.1., Camera: CCD). Considering the three-dimensional nature of cellular architecture including intracellular vesicles a total depth of 10 µm of each field of view was examined with images taken at 1 µm intervals. The 11 images were then stacked using the ‘’Full Focus’’ feature in the BZ-X800 Analyzer Software (version 1.1.2.4 Keyence). Images were analyzed using ImageJ (NIH, Bethesda, MD, USA). After converting the stacked microscopic image to 8-bit greyscale, equal Yen thresholding and subtraction of background was used to quantify the area percentage of the aggresome in each of the images. The values were normalized to the mean of the untreated control (CD34) from each of the three independent experiments. An ordinary two-way analysis of variance (ANOVA) was performed using GraphPad Prism version 10.3.1. Statistical significance was denoted as following (* *p* < 0.05, ns: not significant). Each point on the graph represents an image, the different shades correspond to data points from the three independent experimental replicates, and the mean of each of the individual experiments is plotted as a cross mark (🞫).

#### Immune blotting

Cells grown and differentiated on 35 mm µ-Dish (ibidi^®^) were washed once with PBS, lysed using 200–300 µl of Pierce^®^ RIPA buffer (Thermofisher SCIENTIFIC) containing protease inhibitor (cOmplete ULTRA protease inhibitor - Roche), snap frozen in liquid nitrogen and stored at -80 °C until further utilization. Prior analysis, cell lysates were centrifuged at 4 °C. The protein concentration of the supernatant was determined using the micro-BCA™ protein assay kit (Thermofisher SCIENTIFIC) according to manufacturer´s instructions. Similarly, tissue lysates were prepared from muscle biopsies derived from the two patients carrying the two pathogenic *FLNC* variants of interest as well as tissue lysates from two unaffected controls. The use of patient material for research was performed after ethical approval from the local ethics commission of the Ruhr University Bochum, Medical Faculty (4368-12, May 2012). The lysed muscle tissue was next subjected to sonication to improve lysis (1 min, 1 W, 20% amplitude). The sample was further prepared for electrophoresis by 5 min incubation at 95 °C at a 3:1 ratio with 4x Laemelli buffer with addition of ß-mercaptoethanol as a reducing agent at a final concentration of 5%. 20 µg of protein was loaded in each well of a 4–20% polyacrylamide gel (4–20% Mini-Protean TGX precast, Bio-Rad). PageRuler™ Plus Prestained Protein ladder was used as a marker (#26620, Thermofisher SCIENTIFIC). Four gels were run in parallel using the Mini-PROTEAN Tetra Vertical Electrophoresis Cell chamber (Bio-Rad) using SDS 1x as running buffer. The protocol of SDS-PAGE was as follows: 5 min 40 V, 15 min 100 V, 40 min 140 V. Following gel electrophoresis, an additional gel was fixed and stained with Coomassie brilliant blue (CBB) used for normalization. Protein transfer to a nitrocellulose membrane (GE10600002, Amersham™) was conducted utilizing Towbin buffer in the Trans-Blot^®^ SD Semi-Dry Transfer Cell (Bio-Rad) with a voltage of 12 V for 50–55 min. A ponceau membrane staining was conducted in the immune blotting of tissue lysates to monitor efficient protein transfer and for a total protein normalization to reduce material consumption accompanying CBB staining of an additional gel. Membranes were blocked for 1 h, 4 °C with 5% BSA in TBS-T buffer and incubated with primary antibodies overnight (anti-FLNc: HPA006135 sigma, anti-p62: 56416 abcam, anti-LC3B: 192890 abcam, anti-DNAJC10: 13101-1 proteintech, anti-HYOU1/GRP170 [23]). The next day, membranes were washed thrice with TBS-T for 5 min each and incubated for 1 h with a Horseradish Peroxidase HRP-coupled corresponding secondary antibody. Blots were developed using Radiance Q (Azure biosystems) in the Azure c600 imager. Quantification was made with ImageJ (NIH, Bethesda, MD, USA). An ordinary two-way analysis of variance (ANOVA) was performed using GraphPad Prism version 10.3.1. Statistical significance is denoted as following (* *p* < 0.05, ** *p* < 0.01, *** *p* < 0.001, and **** *p* < 0.0001).

#### Calcium imaging

To monitor changes in [Ca^2+^]_i_, cells were transiently transfected with pcDNA3[Twitch-2B] (kindly provided by Oliver Griesbeck, Addgene plasmid #49531 [24] (0.25 µg per 35 mm µ-Dish-ibidi^®^) by using Lipofectamine (Invitrogen) according to the manufacturer’s instructions. All experiments were performed using single myotubes at ambient temperature. Fluorescence was recorded using an inverted microscope (Zeiss Axiovert 200, Carl Zeiss AG, Göttingen, Germany) equipped with a Zeiss oil immersion objective (100x/1.4), a Polychrome V illumination source and a photodiode-based dual emission photometry system suitable for CFP/YFP-FRET (FEI Munich GmbH, Germany). For FRET measurements, single myotubes were excited at 435 nm wavelength with light pulses of variable duration (20 ms to 50 ms; frequency: 5 Hz) to minimize photobleaching. Corresponding emitted fluorescence from CFP (F_480_ or F_CFP_) or from YFP (F_535_ or F_YFP_) was acquired simultaneously and FRET is defined as the ratio F_YFP_/F_CFP_. Fluorescent signals were recorded and digitized using a commercial hardware/software package (Multi-Channel Data Acquisition Interface ITC-18 with an integrated D/A board and Patchmaster software, both HEKA Elektronik, Germany). All traces were normalized to the initial ratio value before agonist application (normalized FRET = FRET/FRET_0_). Extracellular solution (in mmol/L) was composed as follows: NaCl 122, KCl 5.4, CaCl_2_ 0.5, MgCl_2_ 1.0, HEPES/NaOH 10.0, pH 7.4.

### 3D differentiation of iPSC-derived myoblasts (CD82 + Cells)

Musculoids were obtained through embedding single CD82 + cells with Matrigel^®^ in an elastic pocket form of PARAFILM^®^. Each pocket would contain 50 × 10^3^ CD82 + cells within 30 µl of Matrigel^®^. To maintain comparable conditions for the different lines used, a cellular suspension of 50 × 10^3^ cells in 15 µl growth medium was made. The suspension was further mixed with Matrigel^®^ at a 1:1 ratio. The Mixture was incubated for 25 min at 37 °C, 5% CO_2_ in a humidified incubator. After polymerization of the Matrigel^®^, organoids were transferred to growth medium containing-pHEMA-coated 24-well plate wherein each organoid was placed in a single well of the plate. Growth medium was replaced every second or third day for six-seven days to be replaced with DMEM: F12 media supplemented with non-essential amino acids, L + Glutamine, N-2 Supplement and 1X ITS-X (Gibco™). Table 3. provides detailed information about passage number for each of the differentiation experiments.

#### Transmission electron microscopy

For transmission electron microscopy (TEM), skeletal musculoids were fixed over night at 4 °C in 2.5% glutaraldehyde (Sigma-Aldrich) in 0.1 M cacodylate buffer. Musculoids were further washed thrice with 0.1 M Epon-PBS and processed with a tissue processor with 1% osmium tetroxide (Leica EM TP). Samples were dehydrated by immersion in increasing ethanol concentrations (25%, 35%, 50%, 70%, 75%, 85%, 100%) and afterwards infiltrated with a propylene oxide and a propylene oxide/resin mixture (Agar 100 Resin Kit). Polymerization of the resin was accomplished at 60 °C for 24 h. Semi-thin Sect. (990 nm thickness) were prepared and stained with 1% Richardson to assess overall tissue morphology. Ultrathin Sect. (190 nm thickness) were prepared and placed at 200 mesh copper grids (3.05 mm). Ultrathin sections were subsequently contrasted with EM AC20 (Uranyless/Ultrostain I and 3% lead citrate/Ultrostain II). Samples were examined and photographed using a transmission electron Zeiss microscope, type EM 109 using with a 2 K-CCD-Camera from TRS. For morphometric analysis, a sarcomere is defined as the repeating unit bordered by two adjacent electron-dense z-disk-like structures. Specifically, the length is measured from the center of the electron dense structures to the center of the adjacent one. Measurements took place only in the regions where these repeating units were distinguishable. Examples are provided in Supplementary Fig. 2. Ultrastructural measurements are obtained from two independent musculoids per sample, with each individual measurement treated as an independent observation.

#### Proteomics

##### Sample preparation

The snap frozen musculoids were lysed by adding 200 µl of 50 mM Tris-HCl (pH 7.8) buffer, 5% SDS containing cOmplete ULTRA protease inhibitor (Roche) using the Bioruptor^®^ (Diagenode) for 10 min (30 s on, 30 s off, 10 cycles) at 4 °C, followed by centrifugation at 4 °C and 20,000 xg for 15 min. A BCA assay was used to determine the protein concentration in the supernatant according to the manufacturer’s instructions. Free sulfhydryl bonds were alkylated with 15 mM IAA at room temperature in the dark for 30 min after disulfide bonds were reduced with 10 mM TCEP at 37 °C for 30 min. Proteolysis was performed on 100 µg of protein from each sample using the S-Trap procedure (Protifi) with a protein to trypsin ratio of 20:1. The trypsin incubation period was 2 h at 47 °C. For acidification, FA was used to stop proteolysis (pH < 3.0). After desalting, all proteolytic digests were checked for complete digestion using monolithic column separation (PepSwift monolithic PS-DVB PL-CAP200-PM, Dionex) on an inert Ultimate 3000 HPLC (Dionex, Germering, Germany) by direct injection of 1 µg sample. At a flow rate of 2.2 µL/min and 60 °C, a binary gradient (solvent A: 0.1% TFA, solvent B: 0.08% TFA, 84% ACN) ranging from 5 to 12% B in 5 min and then from 12 to 50% B in 15 min was used. UV traces were taken at a wavelength of 214 nm [25].

##### LC-MS/MS analysis

A total of 1 µg of each peptide sample was separated using an Ultimate 3000 Rapid Separation Liquid Chromatography (RSLC) nano system equipped with a ProFlow flow control device, coupled with a QExactive HF Orbitrap mass spectrometer (Thermo Scientific, Schwerte, Germany). Peptide concentration was performed using a trapping column (Acclaim C18 PepMap100, 100 μm, 2 cm, Thermofisher Scientific, Schwerte, Germany) with 0.1% trifluoroacetic acid (TFA) from Sigma-Aldrich, Hamburg, Germany, at a flow rate of 20 µL/min. This was followed by reversed-phase chromatography (Acclaim C18 PepMap100, 75 μm, 50 cm) using a binary gradient (solvent A: 0.1% formic acid (Sigma-Aldrich, Hamburg, Germany); solvent B: 84% acetonitrile (Sigma-Aldrich, Hamburg, Germany) with 0.1% formic acid). The gradient was set as follows: 5% B for 3 min, a linear increase to 25% over 102 min, further increasing to 33% over 10 min, and a final increase to 95% over 2 min, followed by a decrease to 5% over 5 min.

For MS survey scans, data were acquired in data-dependent acquisition mode (DDA), with full MS scans ranging from 300 to 1600 m/z at a resolution of 60,000, using the polysiloxane ion at 371.10124 m/z as a lock mass. The maximum injection time was set to 120 milliseconds, and the automatic gain control (AGC) is set to 1E6. Fragmentation involved selecting the 15 most intense ions (threshold ion count above 5E3) with a normalized collision energy (nCE) of 27% per cycle, following each survey scan. Fragment ions were recorded at a resolution of 15,000 with an AGC of 5E4 and a maximum injection time of 50 milliseconds. Dynamic exclusion was set to 15 s.

##### Data analysis

All MS raw data were processed using Proteome Discoverer software version 2.5.0.400 (Thermo Scientific, Bremen, Germany) and searched in target/decoy mode against the human UniProt database (www.uniprot.org, downloaded on November 21, 2019) using the MASCOT and Sequest algorithms. Search parameters include precursor and fragment ion tolerances of 10 ppm and 0.02 Da for MS and MS/MS, respectively. Trypsin was specified as the enzyme, allowing up to 2 missed cleavages. Carbamidomethylation of cysteine was set as a fixed modification, while oxidation of methionine was set as a dynamic modification. Percolator was used to establish a false discovery rate (strict) of 0.01 for both peptide and protein identifications.

A Label-free Quantification (LFQ) analysis was conducted with triplicates for each of the control musculoids and the two *FLNC* mutation-carrying musculoids. Only proteins identified with at least 2 unique peptides were included in the analysis. Percentile was applied, Protein ratio calculation for pairwise comparisons and a t-test (background-based) with a p-value < 0.05 were used to determine significant regulation.

To get a detailed overview of the biological processes, a GO-Term analysis was performed. The online software DAVID (Database for Annotation, Visualization and Integrated Discovery) is used [26, 27] Only significantly dysregulated proteins (p-value of 0.05 or less) with either positive or negative regulation were included in the analysis. GO-Terms were checked for biological process, molecular function and cellular compartment and the results are manually filtered for relevant results.

## Results

### Cells of the myogenic lineage form functional myotubes and exhibit pathological phenotype in the *FLNC* variants in conventional cell culture

**Fig. 1:**
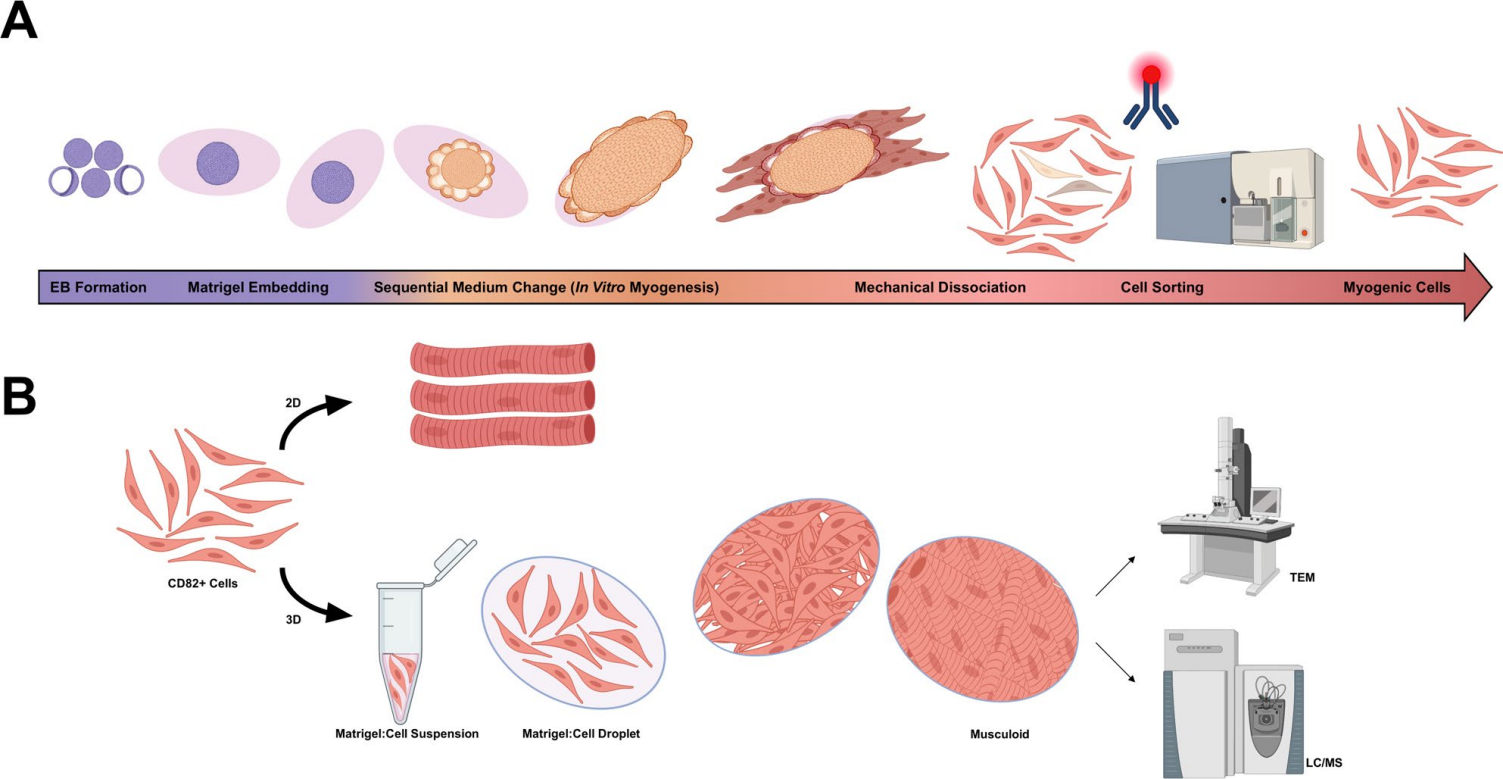
Schematic workflow. **A** Obtaining muscle cells (CD82 + Cells) from iPSCs embryoid bodies (EB) embedded in Matrigel droplet and subjected to different media changes [9] to direct differentiation of the pluripotent stem cells into myogenic lineage through the hSMO model. The hSMOs are dissociated by mechanical pipetting around day 50–60 and the single cells are tagged with CD82 antibodies for myogenic cell enrichment through cell sorting. **B** CD82 + cells are either differentiated in the conventional 2D cell culture into multinucleated myotubes or through a 3D scaffold where the cells are mixed with Matrigel^®^. Droplets of the mixture are incubated at 37 °C for polymerization. Upon confluency of the cells in the droplets, differentiation medium is used. The resulted organoid is referred to as musculoid throughout the manuscript. Around day 35–40 these musculoids are examined for 1- ultrastructural analysis via transmission electron microscopy (TEM) and 2- proteomic profiling with LC-MS/MS

A control iPSC line (CD34) and the two previously generated *FLNC* iPSCs lines were used to form embryoid bodies (EBs) according to the hanging drop method [28]. Sequential introduction of cytokines and growth factors to mimic myogenesis in vitro was conducted following the hSMO model [8, 9]. Around day 50 the organoids start to present myotubes at the periphery of the main structure as well as some bud regions previously described to be rich in PAX7-positive cells [8]. Around that stage, the organoid was mechanically dissociated by forcing it into a 1000 µl pipette tip. For enrichment of the myogenic cells, tagging with a CD82 conjugated antibody was followed by cell sorting (Fig. 1).

**Fig. 2:**
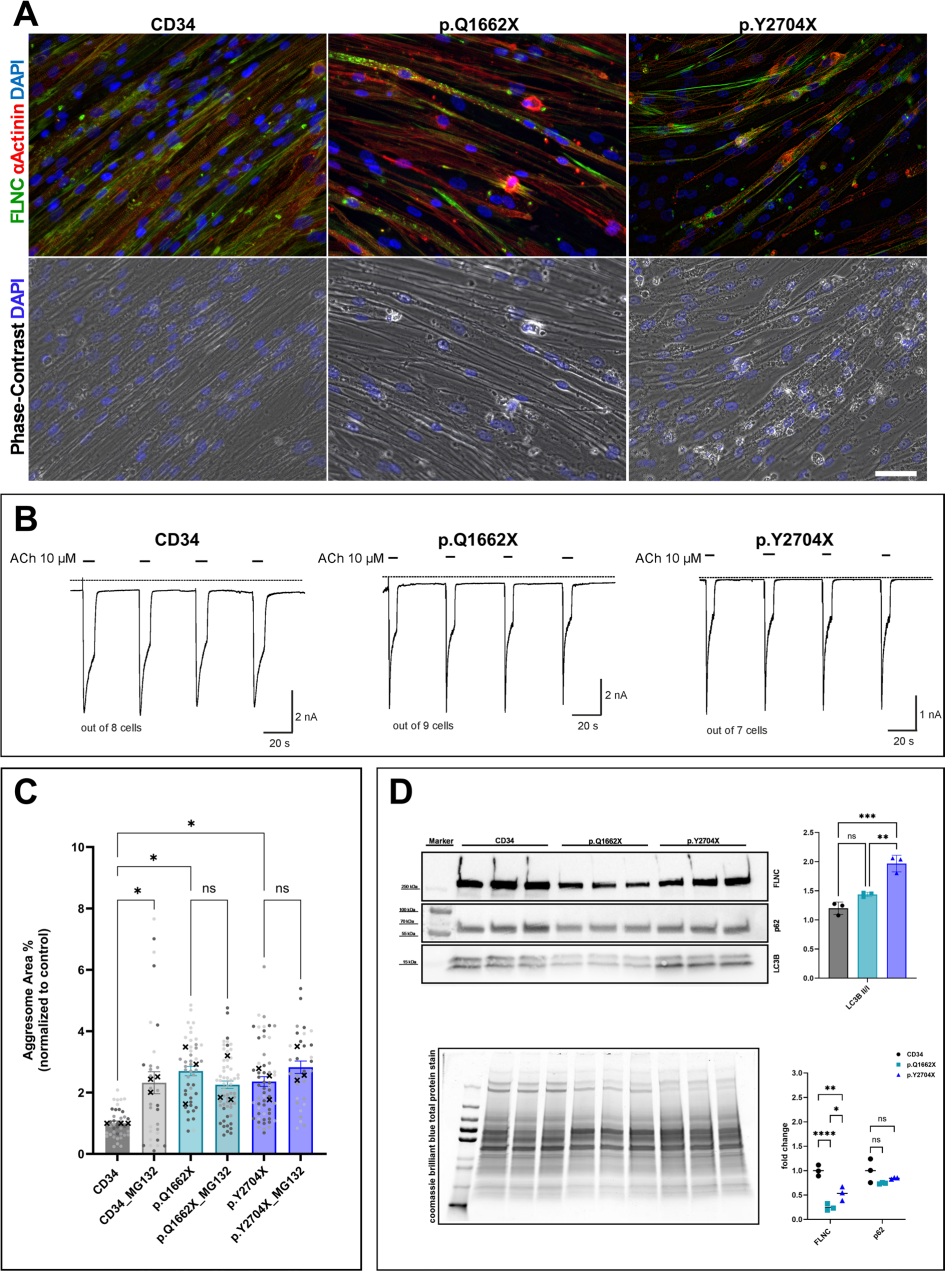
Conventional 2D differentiation of the CD82 + cells.** A** Immunohistochemical staining of 10 days differentiated tubes of a control sample CD34, the p.Q1662X and p.Y2704X *FLNC* variants where anti-FLNc_/_a (RR90) is green, anti αActinin is red and DAPI in blue staining nuclei. The lower panel shows phase-contrast microscopy images with DAPI. Scale bar: 50 μm. **B** Representative recordings of acetylcholine-induced changes in holding current in single myotubes of the three used lines. ACh (10 µM) was applied as indicated by the bars. Holding potential − 90 mV. Dashed line indicates zero current level. **C** Aggresome reactivity as percentage of area in each field of view. The values were normalized to the mean of the untreated control (CD34) from each of the three independent experiments. Each point on the graph represents an image and the different shades correspond to data points arising from three independent experimental replicates and the mean of each of the independent experiments is marked with a cross (🞫). Myotubes were treated with MG132, a proteasomal inhibitor commonly used as a positive control for aggresome formation. An ordinary two-way analysis of variance (ANOVA) is performed using GraphPad Prism version 10.3.1. Statistical significance is denoted as following (ns: not significant, * *p* < 0.05). Error bars indicate SEM. **D** Immunoblotting of three independent 2D-myotubes cultures normalized to a CBB total protein stain showing a decrease in FLNc protein levels, a decrease in p62 protein levels and an increase in the LC3B II/ LC3BI ratio indicating an increased basal autophagic flux in both *FLNC* variants’ myotubes. All independent experiments have been conducted using a single CD82 + population per condition. While CD82 + populations have been propagated from 10–16 independent hSMOs, ANOVA is performed and statistical significance is denoted as following (ns: not significant, * *p* < 0.05, ** *p* < 0.01, *** *p* < 0.001, and **** *p* < 0.0001)

The percentage of the CD82 + population varied between 74% of the total cell number in the p.Q1662X sample to 97% in the control line (Supplementary Fig. 1). This population was assigned the name CD82 + cells and used for conventional two-dimensional cell culture. At about 10 days of differentiation, and especially after medium change, numerous tubes exhibited spontaneous contraction (data not shown). Moreover, striation of the muscle tubes was visible using simple light microscopy (Fig. 2A). Immunohistochemical staining further showed an obvious impairment in the differentiation efficiency of the two variants in comparison to the control line where numerous nuclei have failed to fuse within the α-Actinin/FLNc_/_a stained myotubes (Fig. 2A). Although the immunoreactivity of FLNc_/_a showed multiple lesion-like areas in the myotubes of the CD34 line, this lesion-like reactivity was distributed along the tubes whereas in the two *FLNC* variants these lesions were located along formed myotubes and around nuclei in a speckled manner. Additionally, detachment of multiple tubes in the *FLNC* variants culture was observed.

To assess functional expression of acetylcholine receptors (nAChR) in myotubes, whole-cell patch clamp experiments were performed to measure nAChR currents in response to acetylcholine. As illustrated by the representative current recordings in Fig. 2B, superfusion of a cell with ACh resulted in an inward current that rapidly declined. The amplitude of the acetylcholine-induced inward current remained stable during repetitive applications of ACh. This behavior was found in each of all tested cells by means of this experimental protocol, demonstrating functional sensitivity of the myotubes to ACh and indicating an advanced stage of differentiation.

Since aggregation of the mutant protein is a main pathology in filaminopathies, aggresome formation was assessed using the PROTEOSTAT^®^ Aggresome Detection kit. Quantification of the images showed a statistically significant (*p* < 0.05) increase in aggresome formation by 2.6 and 2.3-fold for the p.Q1662X and p.Y2704X *FLNC* variants respectively (Fig. 2C). Treatment with MG132, a potent and broadly used proteasome inhibitor, increased the aggresome formation in the CD34 line by 2.3-fold whereas in contrast, no statistically significant change was observed in the two *FLNC* variants (Fig. 2C).

Immunoblotting of the conventionally differentiated tubes revealed significantly reduced FLNc protein levels in both p.Q1662X and p.Y2704X myotubes in comparison to the FLNc protein levels detected in myogenic cells derived from the control line CD34 (Fig. 2D). Additionally, an increased basal autophagy flux was observed with reduced p62 levels and an elevation in LC3 II to LC3 I ratios in both *FLNC* variants’ samples although to different extents.

Altogether, the model successfully resulted in differentiation of iPSCs into myogenic cells that can form functional tubes in conventional 2D cell culture and exhibit a pathological phenotype mirroring hallmarks described in muscle biopsy specimen of these patients as described before [14].

### Ultrastructural findings in the musculoid of the *FLNC* variants showed MFM pathology

**Fig. 3:**
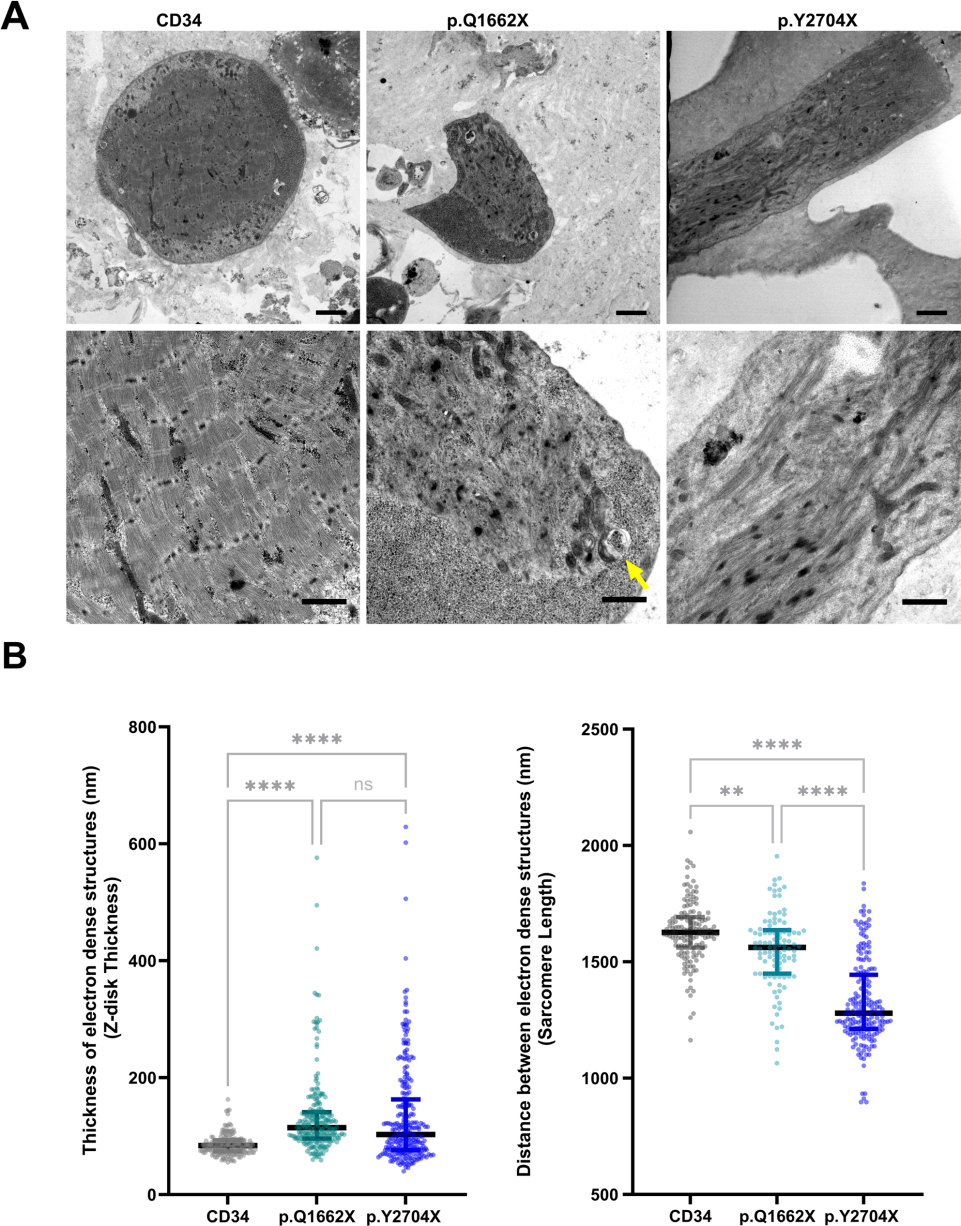
TEM of 3D musculoids. **A** Upper panel showing an overview of each of the three musculoids’ section reflecting the disorganization accompanied with both p.Q1662X and p.Y2704X samples in comparison to the control musculoid CD34. Scale bar: 2500 nm. A higher magnification in the lower panel demonstrates thickening of z-disks in both *FLNC* variant musculoids. A multimembrane vesicle (autophagosome) is shown in the p.Q1662X sample (lower panel, arrow). Scale bar: 1000 nm. **B** Quantification of z-disk thickness and sarcomeric length where each point represents a single measurement. Bars represent medians and quantiles. An ordinary one-way analysis of variance (ANOVA) is performed using GraphPad Prism version 10.3.1. Statistical significance is denoted as following (* *p* < 0.05, ** *p* < 0.01, *** *p* < 0.001, and **** *p* < 0.0001). Significance annotations are displayed in “grey”, indicating that results require cautious interpretation due to limited biological replication and potential pseudoreplication

Ultrastructural examination of CD82 + 3D musculoids revealed perturbed organization of myofibrils in mutant *FLNC* musculoids as seen in (Fig. 3A). However, sarcomere formation in all three samples was observed. Measurement of sarcomeres in 26 field of view of CD34 musculoids, 49 fields of view of p.Q1662X musculoids, and 27 field of view of p.Y2704X musculoids showed a significant decrease in sarcomeric length in both *FLNC* mutant musculoids in comparison to the sarcomeres formed in the control line. Notably, sarcomeres formed in p.Y2704X are further shortened compared to those in p.Q1662X variant musculoid (*p* < 0.0001). Given FLNc’s role as a pivotal z-disk structural protein, a total of 179, 222 and 257 z-disks in CD34, p.Q1662X, and p.Y2704X musculoids were analysed reavealing a significant increase in the z-disk thickness in both *FLNC* mutant samples relative to the control (*p* < 0.0001) and no significant difference between the two pathogenic *FLNC* mutations. Quantifications are shown in (Fig. 3B). Z-disk streaming is observed in various areas of the musculoid sections of the two FLNC mutant samples (arrowheads - Supplementary Fig. 2). Events of autolysosome formation were captured where cargo filled autophagosomes are about to fuse with lysosomes and are shown in Supplementary Fig. 2 (asterix). Further subcellular features observed are also shown in Supplementary Fig. 2 (mitochondria, lysosomes, multimembrane vesicles, nuclei and sarcomeres).

### Proteomic analysis allowed for the identification of novel disease-associated proteins involved in ER stress and protein folding

**Fig. 4:**
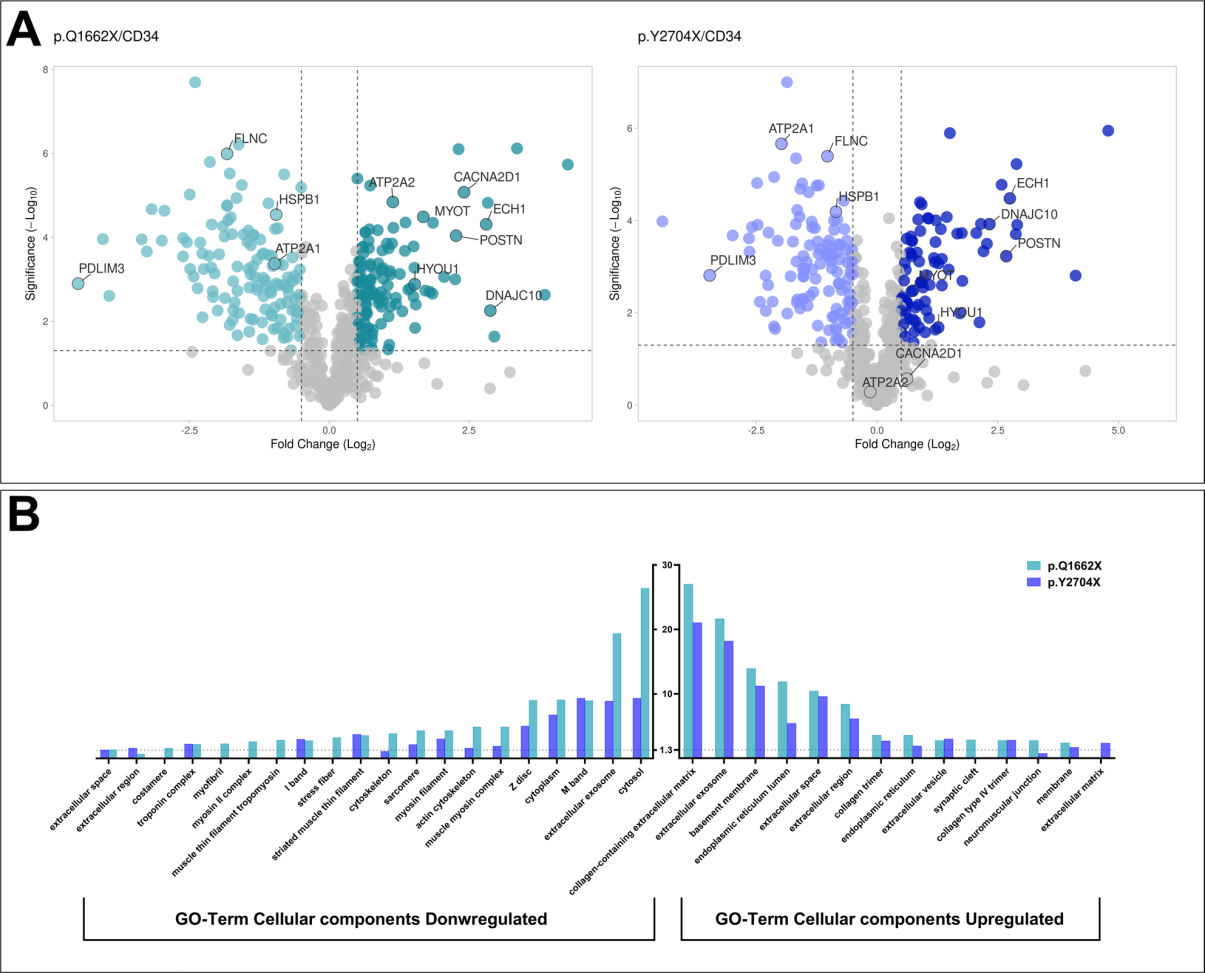
Proteomic analysis summary. **A** Volcano plots displaying differentially abundant proteins in p.Q1662X musculoids in comparison to CD34 (pQ1662X/CD34) and p.Y2704X in comparison to CD34 (p.Y2704X/ CD34) [29]. Whereas lighter green or blue dots represent proteins with decreased abundance, the darker green and blue dots represent proteins with increased abundance in the mutant musculoids. **B** Comparative visualization of GO-term based results of dysregulated proteins in both musculoid models

Proteome-wide comparisons of CD34 and *FLNC* p.Q1662X musculoids revealed 151 proteins (25%) that are differentially expressed (DE: fold-change ≤ 0,5 (downregulated) or ≥ 2 (upregulated), p-Value ≤ 0.05). Whereas a comparison of CD34 and *FLNC* p.Y2704X musculoids revealed a dysregulation of 123 proteins (20%) (Fig. 4A). The GO-Term analysis for biological processes shows that cell adhesion and collagen fibril organization are significantly upregulated processes in addition to alternative mRNA splicing affecting both *FLNC* variants. Of note, sarcomere organization represents the most significantly reduced process in both samples. In addition to the upregulation of extracellular structures of the cellular component GO-Term analysis, a significant upregulation of the endoplasmic reticulum ER-associated components is observed. However, this dysregulation is more prominent in the p.Q1662X variant (Fig. 4B). Relevant commonly dysregulated proteins are highlighted in the volcano plots of (Fig. 4A). Notably, DNAJC10 the ER-resident heat shock protein Hsp40 is significantly upregulated in both *FLNC* variants, with a 7-fold increase in p.Q1662X, 5-fold increase in p.Y2704X compared to CD34 controls. Additionally, GRP170 (HYOU1), a chaperone critical for protein folding and ER stress response is commonly upregulated in both variants relatively to control musculoids (2.8-fold and 2.4-fold increase in p.Q1662X and p.Y2704X respectively). Altogether, proteomic findings indicate impaired differentiation marked by reduced FLNc expression and key cytoskeletal structures to muscle cells, while also highlighting novel disease-associated proteins involved in ER stress and protein folding.

**Fig. 5:**
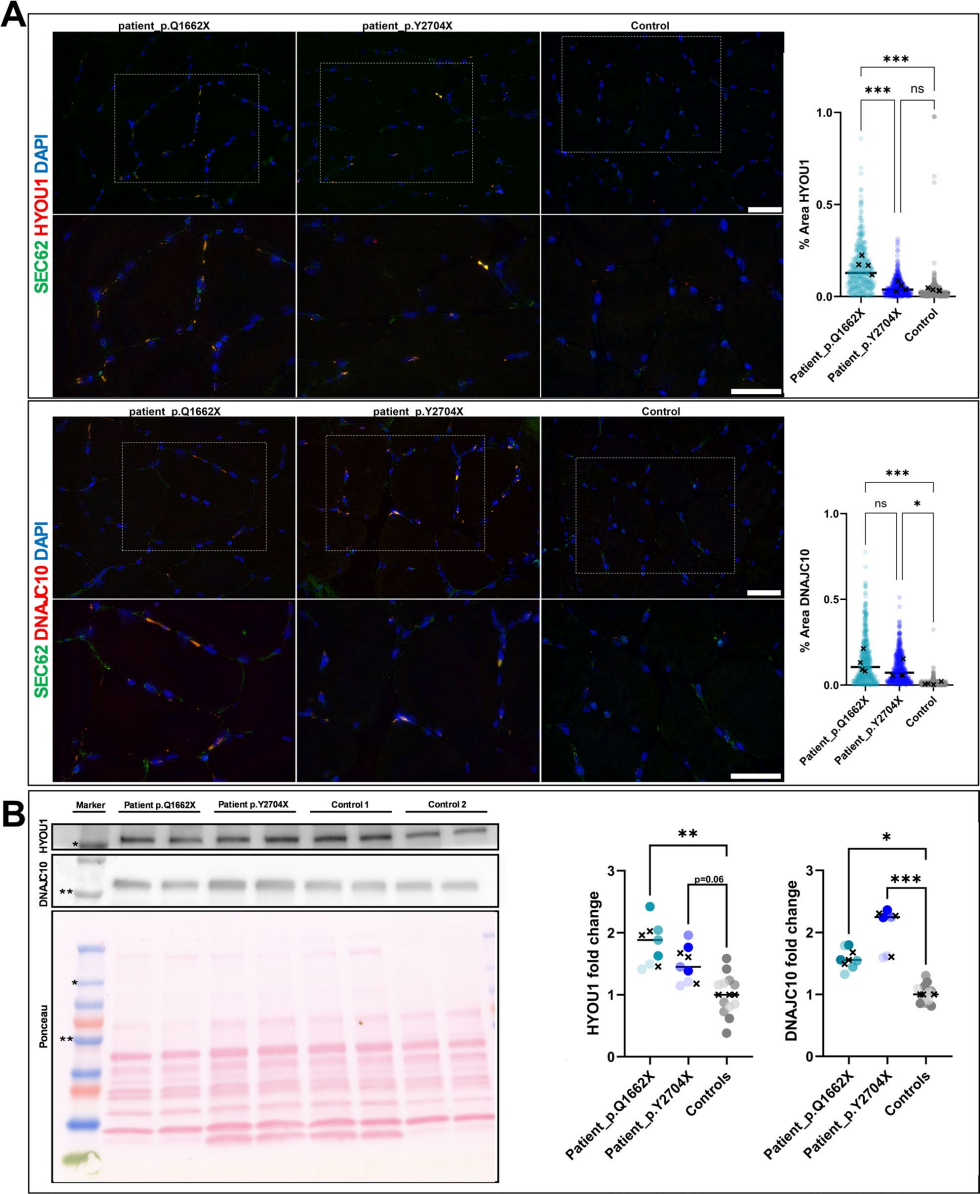
Validation of proteomic data in patient biopsies.** A** Fluoresce-based immunostainings with quantification of HYOU1 (upper panel) and DNAJC10 (lower panel). Each dot represents a microscopic field of view. Sections are co-stained with SEC62 confirming ER localization. **B** Immunoblotting of biopsy lysates normalized to a ponceau total protein stain. Each dot represents the relative densitometric value of bands from three independent experiments, each made with technical duplicates and two different control biopsies. Different shades indicate data arising from independent experiments. **A**, **B** The mean of individual experiments is plotted as a cross mark (🞫) and an ANOVA is performed using GraphPad Prism version 10.3.1. Statistical significance is denoted as following (ns/value: not significant, * *p* < 0.05, ** *p* < 0.01, and *** *p* < 0.001)

To validate these findings, muscle biopsies from the same patients from whom the *FLNC* mutant cell lines were derived (p.Q1662X and p.Y2704X) were analysed, alongside biopsies from two control individuals (unaffected, age-matched). Immunohistochemical staining of HYOU1, depicted in Fig. 5A, revealed a granular staining pattern in perinuclear regions in patient_p.Q1662X with a markedly reduced reactivity in patient_p.Y2704X and a barely visible signal in control. Quantifications across 400 fields of view per biopsy demonstrated 0.171% of area to be stained in the biopsy of the *FLNC* patient harbouring the p.Q1662X variant. An area of 0.052% in *FLNC* patient harbouring the p.Y2704X variant and an area of 0.036% in the unaffected control biopsy were detected. A similar pattern of staining (granular perinuclear) was detected for DNAJC10. However, quantifications of 525 fields of view show a significantly enhanced signal in both patient biopsies in comparison to control (Fig. 5A). Sections were co-stained for SEC62, confirming ER co-localization.

To further corroborate proteomic findings, immunoblotting was performed and revealed an increase in HYOU1 expression in protein extracts of patient biopsies, with 1.81-fold in p.Q1662X and 1.48-fold in p.Y2704X compared to controls. Additionally, increased DNAJC10 levels were also verified with 1.57-fold increase in p.Q1662X and 2.05-fold in p.Y2704X muscle biopsies. Data were collected from three independent experiments, each performed in duplicates (Fig. 5B).

Consistent with biopsy data, 2D myotube immunoblotting showed significantly increased DNAJC10 (1.85-fold in p.Q1662X; 2.14-fold in p.Y2704X) and HYOU1 (2.05-fold in p.Q1662X, though not in p.Y2704X), suggesting that our 2D myotubes may suffice for studying early molecular alternations in filaminopathies (Supplementary Fig. 4).

Both, results of immunostaining and immunoblotting align with proteomic findings related to ER stress-associated proteins (HYOU1 & DNAJC10).

**Fig. 6:**
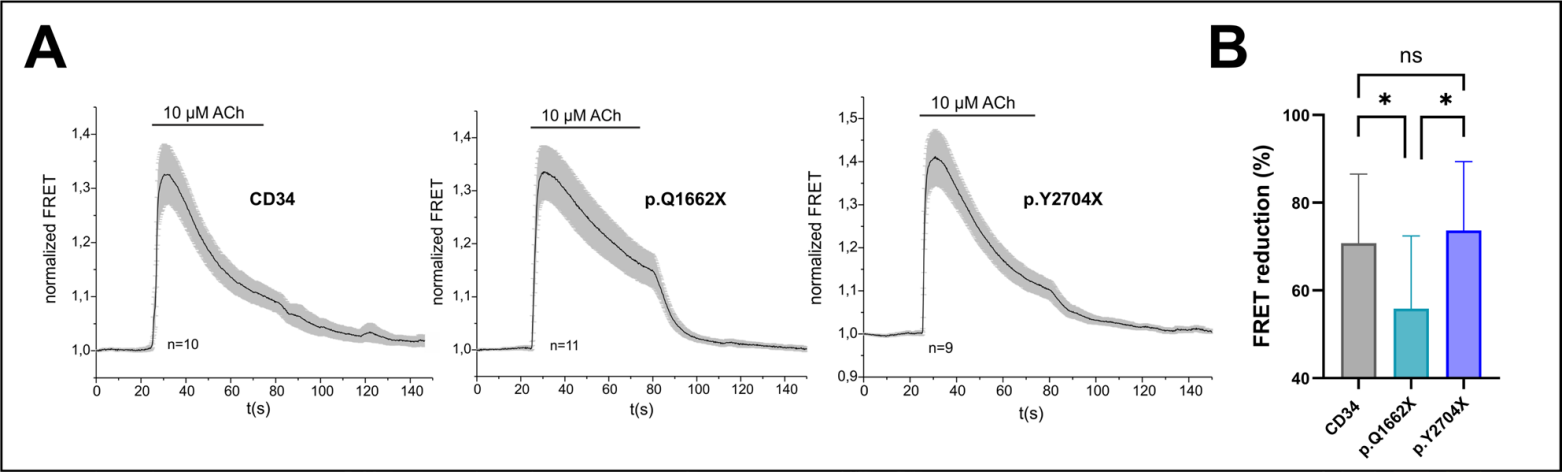
Calcium Imaging.** A** Summarized FRET-recordings from 2D-differentiated myotubes transfected with Twitch2B to monitor the increase in [Ca^2+^]_i_ during exposure to ACh using the control line CD34 and the two *FLNC* p.Q1662X and p.Y2704X myotubes. Application of ACh (10 µM) as indicated by the bars. All recordings are illustrated as mean values (black curve) ± S.E (shaded in grey). Number of experiments as indicated. **B** Summarized data of the decay of [Ca^2+^]_i_-transients during acetylcholine application (determined by the ratio FRET_50s after peak_/FRET_peak_). After testing normal distribution and equal variance, Student´s t-test was used for comparing the decline of the FRET signals. P-values less than 0.05 were considered statistically significant and marked by an asterisk, ns: not significant

The mass spectrometric results also highlighted dysregulation in calcium related proteins, a pathway critically linked to ER function. Most notably, CACNA2D1 -a voltage-gated calcium channel subunit- is significantly upregulated with 5.3-fold in p.Q1662X musculoid relative to control. Concurrently, ATP2A1 (SERCA1) is downregulated in both *FLNC* variants’ musculoids. However, an increase of ATP2A2 (SERCA2) is observed in p.Q1662X and not in p.Y2704X musculoids (Fig. 4A). To functionally validate these measurements, FRET-based calcium imaging in 2D-differentiated myotubes was carried out. The efficiency of nAChR to induce changes in [Ca^2+^]_i_ in myotubes was investigated using a fluorescent biosensor for monitoring changes in cytosolic Ca^2+^ (Twitch-2B [24]), The Twitch-2B biosensor responds to Ca^2+^-binding with an increase of the FRET ratio and contains a calcium binding motif based on the C-terminal domain of *Opsanus tau* Troponin C framed by a donor and an acceptor fluorescent protein. As shown in the summarized FRET recordings in Fig. 6, application of ACh (10 µM) caused a rapid increase in FRET ratio that gradually declined in the presence of ACh. The decline of [Ca^2+^]_i_-transients during stimulation of nicotinergic receptors partially reflects desensitization of nAChRs, but is also be determined by the time courses of Ca^2+^ extrusion and sarcolemmal Ca^2+^ removal. Interestingly, the decline of [Ca^2+^]_i_-transients in p.Q1662X was significantly slower than its counterparts of both CD34 and p.Y2704X myotubes. To evaluate the reduction of [Ca^2+^]_i_-transients in the presence of ACh, we evaluated the ratio FRET_50s after peak_/FRET_peak_ for each single experiment. The summarized data shown in Fig. 6 indicate a more than 70% reduction of the FRET signal during continuous ACh-stimulation in CD34 and p.Y2704X tubes compared to about 55% in p.Q1662X mutant myotubes. These data suggest that sarcolemmal Ca^2+^ removal is impeded in the p.Q1662X variant.

## Discussion

Recent advancements in skeletal muscle differentiation protocols have enabled the generation of various in vitro myogenesis models from iPSCs. Among these approaches, the hSMO protocol results in PAX7 + populations preserved in their quiescent state [18]. Moreover, the CD82 tagging was used for enrichment of myogenic cells based on reports highlighting its role as a marker for human muscle satellite cells [30]. Guided by these advancements, we used previously propagated iPSC lines from patients carrying two novel pathogenic *FLNC* variants [14, 16, 17] and a control line [20] to obtain cells of the myogenic lineage. We successfully obtained cells positive for CD82 with an efficiency ranging between 74% and 97% in the different lines used.

Since preserving the obtained myogenic cells in their quiescent state was not of relevance for modelling a late-onset disorder like filaminopathies, we have expanded these cellular populations using commercially available human skeletal muscle growth media. Following conventional 2D cell culture differentiation protocols, the CD82 + cells were differentiated into multinucleated myotubes. Furthermore, cross striation was confirmed by α-actinin immunohistochemical staining in numerous myotubes, however to different extents. Both *FLNC* variant myotubes exhibited impaired differentiation marked by thinner tubes, a profusion of unfused nuclei and a speckled dot-like reactivity to the RR90 anti-FLNc_/_a antibody [21]. FLNc has been reported to have key roles in early injury response [31], muscle repair [32] and myofibrillogenesis [33]. Therefore, there must be a distinction between longitudinal immune reactivity that emerges from the dynamic nature of FLNc and its role in differentiation, its localization at cross striation and the speckled dot-like distribution that likely emerges from aggregating of mutant proteins soon after translation. Moreover, the functionality of the resulting myotubes has been validated by current measurements following exposure to acetylcholine indicating the expression of nACh receptors which marks a terminally differentiated functional myotube [34].

Protein accumulation and aggregation is considered as a pathobiochemical hallmark of filaminopathies [12, 13, 35], leading to altered proteostasis and dysregulated protein quality control pathways. Proteostatic stress was evident in both *FLNC* mutant myotubes with basal aggresome formation levels exceeding those in control line. While the inhibition of proteasome through MG132 treatment significantly increased the aggresome formation in control myotubes, it failed to further enhance aggresome levels in the pathological variants (p.Q1662X and p.Y2704X). This suggests that the proteasome system in the cells expressing the pathogenic *FLNC* variants is already overloaded. Additionally, since MG132 inhibits proteasomal activity through binding on the β5, β1 and β2 subunits of the 20 S proteasome [36], one might speculate that the two FLNc protein variants activate other proteasomal units that are beyond the inhibition of MG132 (like, ECM29, PSMF, PA28αβ, PA28γ or PA200 complexes). Notably, our LC-MS/MS measurements unveiled a significant minor upregulation in PSMA2 (1.33-fold in p.Q1662X and 1.64-fold in pY2704X relative to control musculoids) whereby, PSMA2 is an α-subunit and therefore, not a direct target of MG132. In addition, activation of autophagy in both pathological *FLNC* myotubes is marked by reduced p62 levels and elevated LC3B-II/LC3B-I ratios since lysosomal degradation of autophagosome leads to a decrease in p62 levels during autophagy and the conversion of LC3B-I to LC3B-II marks autophagosome formation [37]. For future studies, using other lysis buffers may enable a more precise detection of biological alterations regarding sarcomeric proteins, while definitive characterization of autophagy would require experiments utilizing specific inducers and inhibitors. Altogether, the quantifiable dysregulation of proteasome activity, aggresome formation and the increased basal autophagy flux establish this model as a suitable in vitro platform for screening of possible therapeutic interventions targeting the protein quality and clearance machinery.

To enable ultrastructural analysis of the z-disk pathology at the highest resolution possible and to mimic native muscle structure, CD82 + cells were encapsulated within natural hydrogel droplets (Matrigel^®^), establishing a 3D environment that supports cellular alignment. The cells have differentiated within the Matrigel and exhibited spontaneous contraction that was visible at the periphery of the spherical musculoids (control and filaminopathy models alike). Transmission electron microscopy of control musculoids revealed a fine structural organization of myofibrils with z-disks averaging 78.9 nm (range: 55–111 nm), consistent with established z-disk thickness in healthy human striated muscle (30–100 nm) [10]. In contrast, pathogenic *FLNC* variants p.Q1662X and p.Y2704X both exhibited pronounced ultrastructural alterations with increased z-disk thickness of 119.6 nm (range: 60–295 nm) and 123.7 nm (range: 46–350 nm) respectively, demonstrating z-disks pathology. This observation aligns with another hallmark of filaminopathies, namely z-disk lesions [12]. Notably, the p.Q1662X variant -reported to act via haploinsufficiency- induced aggregates and z-disk lesions, verifying FLNc’s role in maintaining z-disk integrity and homeostasis. This mirrors previously reported observations in Nebulin-KO mice, where the loss of this actin-binding protein similarly affects z-disk structures resulting in significantly thicker z-disks in extensor digitorum longus and soleus muscle fibers [38]. These structural alterations were also confirmed by a significant shortening of sarcomeres in both p.Q1662X and p.Y2704X variants. Such shortness of sarcomere has not been previously directly discussed in filaminopathies. However, in mutant mice harboring a *FLNC* variant analogous to the human p.W2710X mutation, a distance of 25 μm contains 11 z-disks (10 sarcomeres) whereas in WT mice the same distance contain 8 z-disks (7 sarcomeres) [39]. Reflecting that shortened sarcomeric length may be a consistent observation with *FLNC* variants and expanding current knowledge of pathomorphological features associated with this rare form of myopathies. It is important to acknowledge a methodological limitation: sarcomeric measurements were obtained from two independent musculoids per sample and treated as independent observations. This limited biological replication renders the analysis statistically underpowered, and the findings although consistent with known pathology should be interpreted with caution. Future studies must incorporate a larger number of biological replicates to increase statistical power and strengthen the reliability of conclusions. Ultrastructural analysis also revealed autophagic structures as multimembrane vesicle and lysosomes that can be utilized for a prospective quantitative study. Taken together, the musculoid constructs are very suitable for morphometric analysis of skeletal muscle models.

Proteomic analysis of the musculoids unveiled novel molecular contributors related to ER-stress and protein folding that were previously unrecognized in filaminopathies’s pathogenesis, particularly implicating ER-stress and protein folding dysregulations as central mechanisms. Of interest were DNAJC10 and HYOU1. DNAJC10 (Hsp40) an ER-resident disulfide reductase involved in the correct folding of proteins and the degradation of misfolded proteins [40] was significantly upregulated in both *FLNC* variant musculoids. This chaperone is also known to promote apoptotic signaling pathway in response to ER-stress [41]. This was validated in corresponding muscle biopsies of the patients in relation to control biopsies with means of immunohistochemistry and immunoblotting. HYOU1 (also known as GRP170) a member of the Hsp70 superfamily that is believed to interact with unfolded proteins in the ER [42] was also increased in patient derived biopsies confirming the suitability of our musculoids to study molecular processes reflecting pathophysiologies occurring in the complex tissue. Contrary to DNAJC10, HYOU1 has known anti apoptotic effects [43]. We speculate that the upregulation of HYOU1 is a protective response to ER-stress triggered by accumulation of misfolded or aggregating protein. This assumption is supported by the observed activation of autophagy as a cellular attempt to compensate the build-up of toxic protein aggregates. While the precise mechanism that elucidates the role of the two ER-resident chaperones in filaminopathy is yet to be further investigated, the translational relevance of the model underscores its fidelity and utility for studying neuromuscular disorders that originate from muscle. Furthermore, we speculate that the absence of other cellular components in the musculoid -like immune cells and vascularization- may contribute to the entrapment of apoptotic cells leading to impaired differentiation. Prospectively, the CD82 + cells may be utilized in the future in more sophisticated models that better mirror the native muscle’s complexity.

Acknowledging the likelihood of variant-specific pathomechanisms [12, 14], our proteomic analysis of musculoids resembling two different genotypes of filaminopathies further indicated a dysregulation in calcium related proteins unique to the p.Q1662X variant in comparison to both the control and the p.Y2704X variant. This was supported by the results of our FRET Ca^2+^ imaging, functionally exhibiting a significantly slower Ca^2+^ reuptake into the ER after excitation in the 2D myotubes of the *FLNC* p.Q1662X variant.

While acknowledging that the fetal-like characteristics of myogenic progenitors derived from the hSMO model limit their capacity to model adult tissue contexts, both 2D and 3D systems recapitulated filaminopathy pathology. In affected individuals, this pathology may be masked by multicellular interplay leading to a late-onset of clinical manifestation. Nevertheless, prospective studies must aim at achieving higher maturity levels of cellular models reaching beyond fetal stages. Additionally, both the 2D myotubes and the 3D musculoids can be used not only for investigative studies of filaminopathies or other neuromuscular disorders, but also as platforms for therapy screening with possible quantitative readouts. Conclusive findings regarding the pathomechanisms accompanying the mutations investigated remain challenging to establish, due to the absence of biological replicates. Moreover, to distinguish mutation-specific effects from patient-specific background variables, isogenic controls and additional cell lines carrying the same mutations should be studied. This limitation must be addressed in future work. Prospectively, iPSCs or propagated CD82 + cells can undergo gene correction for potential autologous cellular transplantation therapies upon ethical approval.

## Conclusion

Our study employed the hSMO model to generate cells of the myogenic lineage from two iPSCs lines carrying distinct protein-truncating *FLNC* variants. The generated cells have mirrored key aspects of filaminopathies, in both 2D and 3D systems. They have further enabled the identification of novel players in the pathology of the disease that was corroborated by validation is tissue samples from affected individuals. This establishes the model as a robust platform for in vitro studies of filaminopathies and potentially other neuromuscular disorders. However, conclusive unraveling of the pathomechanisms underlying the investigated *FLNC* variants remains elusive.

## Supplementary Information

Supplementary Material 1: Supplementary Fig. 1. Cell Sorting of cells of the dissociated hSMO for myogenic enrichments of CD82 + cells. 10–16 hSMO of each cell line were mechanically dissociated. Dissociated cells were cultivated for 2–3 days prior to myogenic enrichment using a conjugated anti-CD82 antibody. Shortly prior to cell sorting, a sample of each of the populations (CD34, p.Q1662X and p.Y2704X) was stained with DAPI to set the gating strategy to exclude dead cells (first row each). Cellular populations were incubated with the anti-CD82 antibody for myogenic enrichment and briefly before sorting, DAPI was added to the samples (second row each). First, samples were plotted side versus forward scatter (SSC vs. FSC) to identify the cells of interest based on their size and granularity (left). Next, DAPI positive cells were excluded from the analysis (center). At last, CD82 positive populations were selected (right). These populations-referred to as CD82 + cells- were sorted and used for further experiments. Supplementary Fig. 2. TEM of musculoids. (A) p.Q1662X variant, (B) p.Y2704X variant. Both variants exhibit marked sarcomeric disorganization. Relevant cellular organelles and structural features are seen and indicated in both panels including: Z-disk (z) alterations: arrow heads, autolysosome formation: asterisk, lysosome: L, mitochondria: M, sarcomeres: S, nucleus: N, multimembrane vesicle: AV, cross section of myofibrils: rectangle. White bar: 250 nm, Black bar: 500 nm. Supplementary Fig. 3. Individual current measurements. Individual current recordings of acetylcholine-induced changes in holding current in all myotubes measured (cells). Ach (10 µM) was applied as indicated by the bars. Holding potential − 90 mV. Dashed line indicates zero current level. The cell (myotube) marked by an asterisk correspond to the current recording shown in Fig. 2. Supplementary Fig. 4. Immunoblotting of HYOU1 and DNAJC10 in 2D differentiated myotubes. Three independent 2D-myotube cultures were lysed, blotted and normalized to a ponceau total protein stain. An increase of HYOU1 was detectable in p.Q1662X myotubes but not in the p.Y2704X variant. DNAJC10 was significantly increased in both variants’ myotubes. ANOVA is performed and statistical significance is denoted as following (ns: not significant, * *p* < 0.05, ** *p* < 0.01, and *** *p* < 0.001). Supplementary Fig. 5. Representative images of the aggresome assay in 2D myotubes. The left panel shows an overlay of phase-contrast, DAPI (blue) and aggresome staining (red). The middle panel shows the isolated aggresome staining channel. The right panel shows the corresponding binary masks created and used for quantification.

## Data Availability

No datasets were generated or analysed during the current study.

## Abbreviations

ACh: Acetylcholine
EB: Embryoid Body
ER: Endoplasmic Reticulum
FLNc: Filamin C
hSMO: Human Skeletal Muscle Organoid
hiPSCs: Human induced Pluripotency Stem Cells
MFM: Myofibrillar Myopathies
nAChR: Nicotinic Acetylcholine Receptor
PAX7: Paired Box 7
