# Supplementary material for: Unveiling FLNC variants: iPSC-derived myogenic cells as a model to study disease mechanisms
Source: Skelet Muscle. 2026 Feb 12;16:18. doi: 10.1186/s13395-026-00418-5 (PMC13063712; doi:10.1186/s13395-026-00418-5)
Supplement: Supplementary file 1 — Supplementary Material 1: Supplementary Fig. 1. Cell Sorting of cells of the dissociated hSMO for myogenic enrichments of CD82 + cells. 10–16 hSMO of each cell line were mechanically dissociated. Dissociated cells were cultivated for 2–3 days prior to myogenic enrichment using a conjugated anti-CD82 antibody. Shortly prior to cell sorting, a sample of each of the populations (CD34, p.Q1662X and p.Y2704X) was stained with DAPI to set the gating strategy to exclude dead cells (first row each). Cellular populations were incubated with the anti-CD82 antibody for myogenic enrichment and briefly before sorting, DAPI was added to the samples (second row each). First, samples were plotted side versus forward scatter (SSC vs. FSC) to identify the cells of interest based on their size and granularity (left). Next, DAPI positive cells were excluded from the analysis (center). At last, CD82 positive populations were selected (right). These populations-referred to as CD82 + cells- were sorted and used for further experiments. Supplementary Fig. 2. TEM of musculoids. (A) p.Q1662X variant, (B) p.Y2704X variant. Both variants exhibit marked sarcomeric disorganization. Relevant cellular organelles and structural features are seen and indicated in both panels including: Z-disk (z) alterations: arrow heads, autolysosome formation: asterisk, lysosome: L, mitochondria: M, sarcomeres: S, nucleus: N, multimembrane vesicle: AV, cross section of myofibrils: rectangle. White bar: 250 nm, Black bar: 500 nm. Supplementary Fig. 3. Individual current measurements. Individual current recordings of acetylcholine-induced changes in holding current in all myotubes measured (cells). Ach (10 µM) was applied as indicated by the bars. Holding potential − 90 mV. Dashed line indicates zero current level. The cell (myotube) marked by an asterisk correspond to the current recording shown in Fig. 2. Supplementary Fig. 4. Immunoblotting of HYOU1 and DNAJC10 in 2D differentiated myotubes. Three independent 2D- [file 13395_2026_418_MOESM1_ESM.docx]

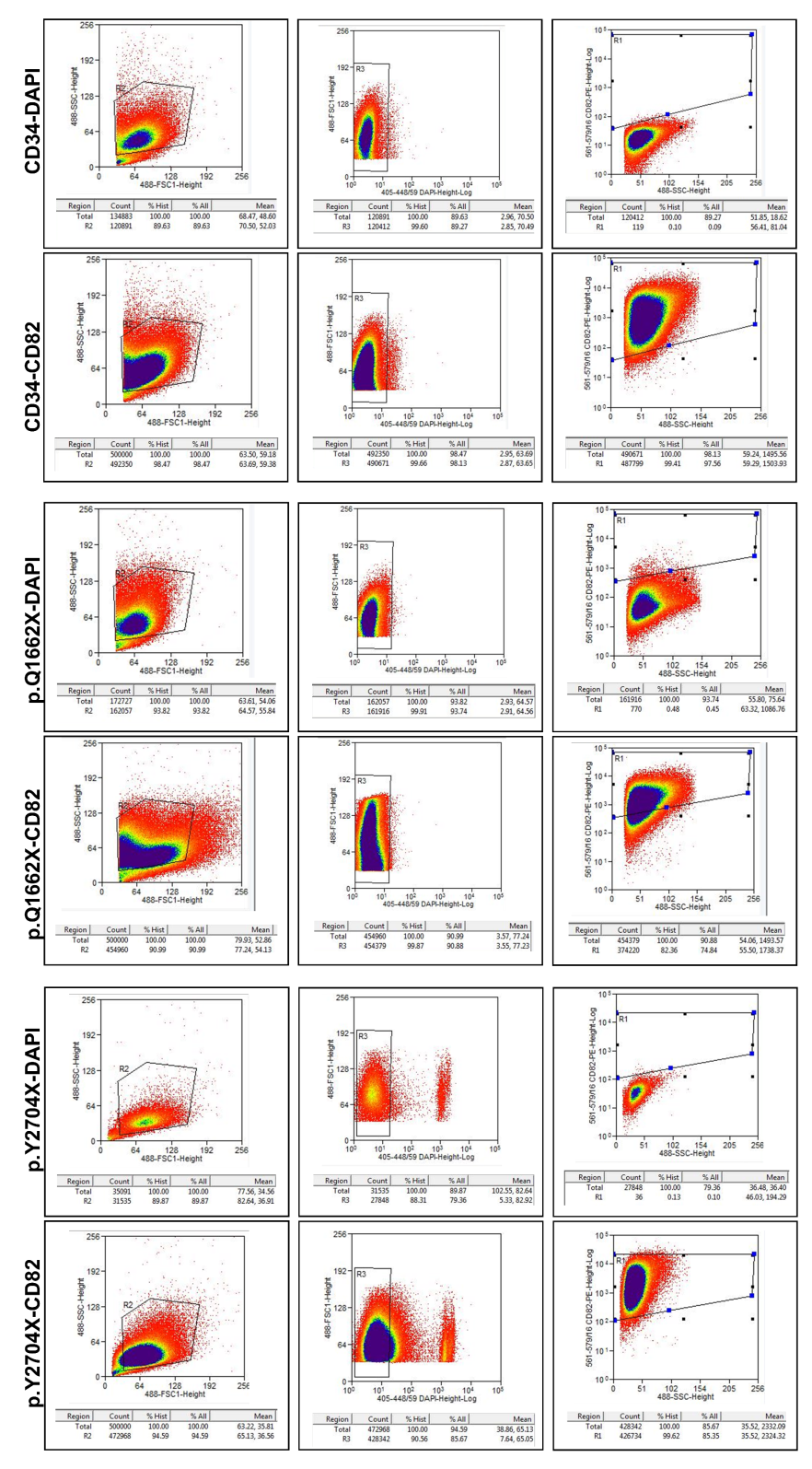


**Supplementary Figure1**. **Cell Sorting of cells of the dissociated hSMO for myogenic enrichments of CD82+ cells**. 10-16 hSMO of each cell line were mechanically dissociated. Dissociated cells were cultivated for 2-3 days prior to myogenic enrichment using a conjugated anti-CD82 antibody. Shortly prior to cell sorting, a sample of each of the populations (CD34, p.Q1662X and p.Y2704X) was stained with DAPI to set the gating strategy to exclude dead cells (first row each). Cellular populations were incubated with the anti-CD82 antibody for myogenic enrichment and briefly before sorting, DAPI was added to the samples (second row each). First, samples were plotted side versus forward scatter (SSC vs. FSC) to identify the cells of interest based on their size and granularity (left). Next, DAPI positive cells were excluded from the analysis (center). At last, CD82 positive populations were selected (right). These populations-referred to as CD82+ cells- were sorted and used for further experiments.


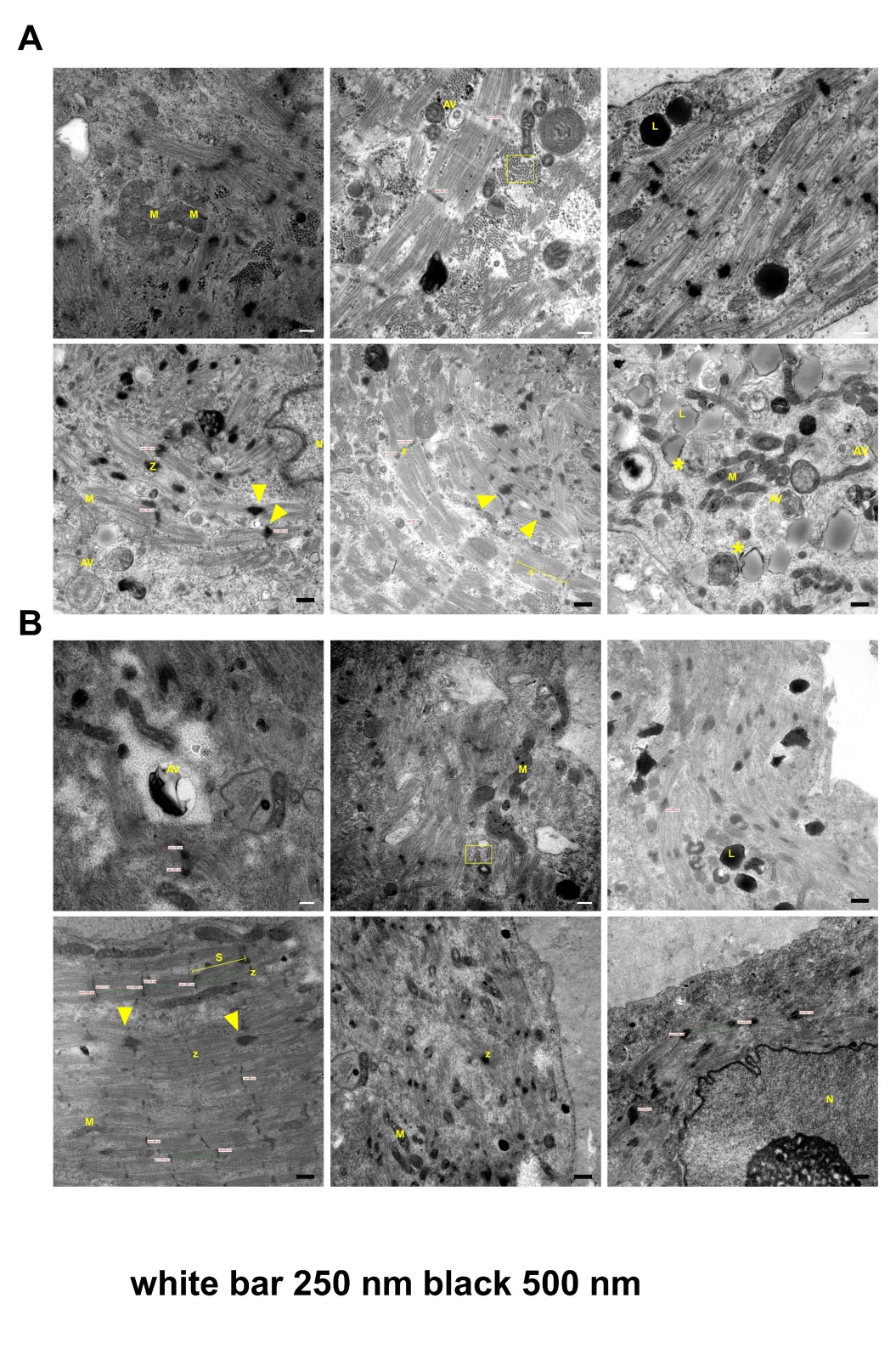


**Supplementary Figure 2. TEM of Musculoids** (**A**) p.Q1662X variant, (**B**) p.Y2704X variant. Both variants exhibit marked sarcomeric disorganization. Relevant cellular organelles and structural features are seen and indicated in both panels including: Z-disk (z) alterations: arrow heads, autolysosome formation: asterisk, lysosome: L, mitochondria: M, sarcomeres: S, nucleus: N, multimembrane vesicle: AV, cross section of myofibrils: rectangle. White bar: 250nm, Black bar: 500 nm.


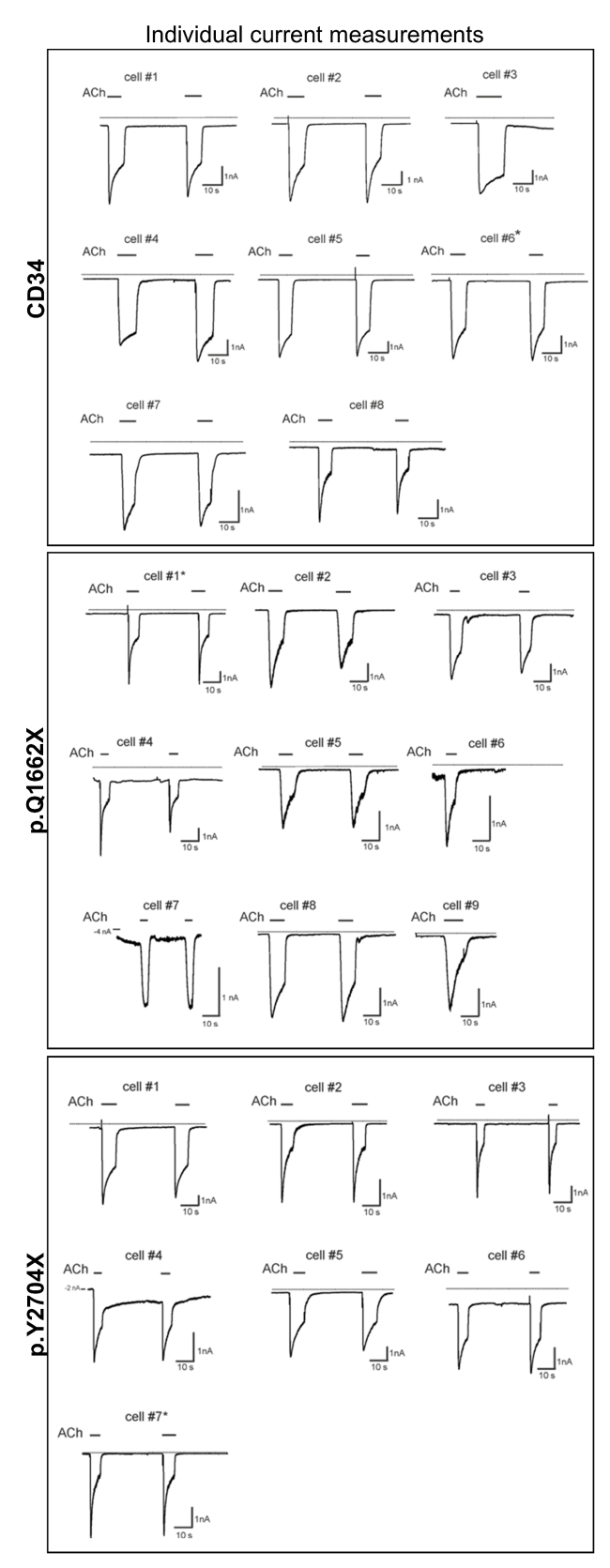


**Supplementary Figure 3. Individual Current Measurements.** Individual current recordings of acetylcholine-induced changes in holding current in all myotubes measured (cells). Ach (10 µM) was applied as indicated by the bars. Holding potential -90 mV. Dashed line indicates zero current level. The cell (myotube) marked by an asterisk correspond to the current recording shown in figure 2.


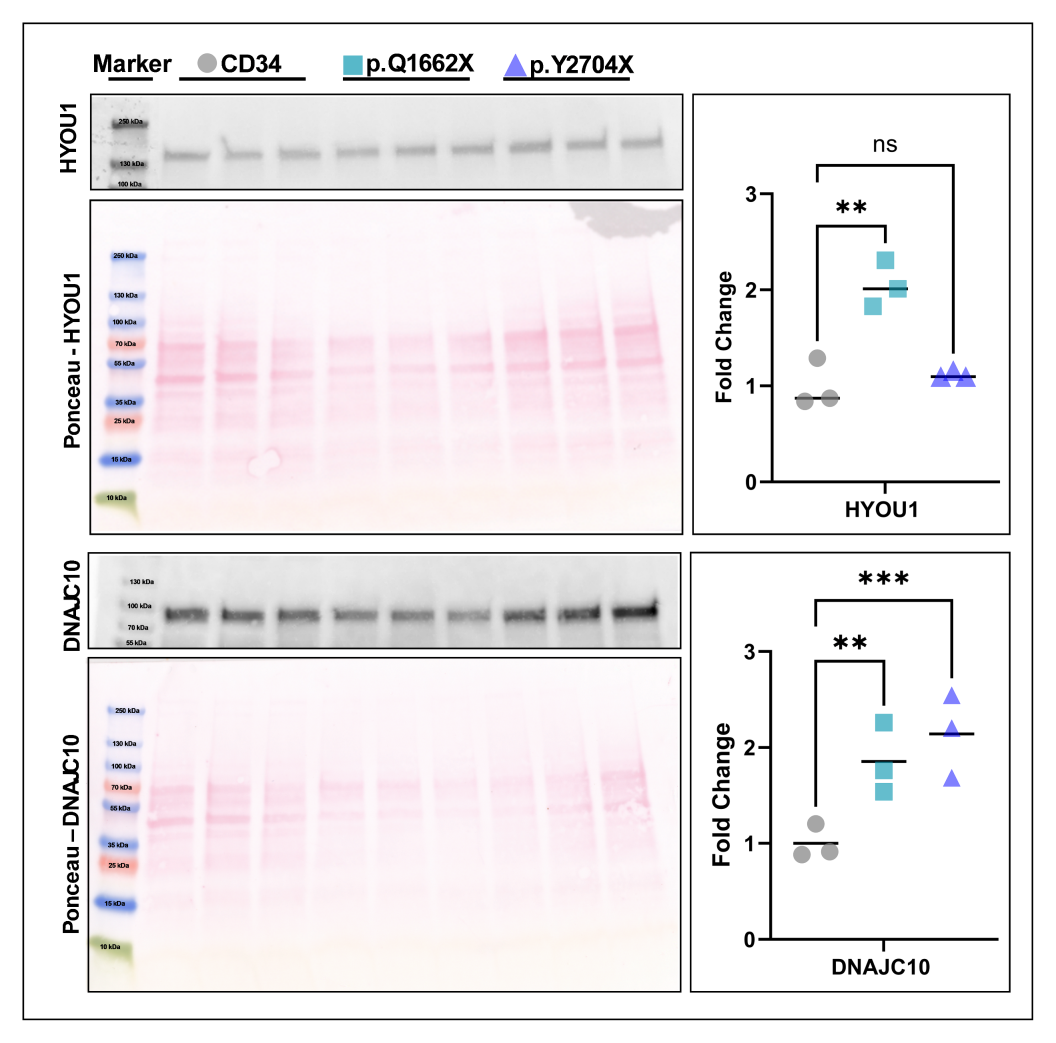


**Supplementary Figure 4. Immunoblotting of HYOU1 and DNAJC10 in 2D differentiated myotubes.** Three independent 2D-myotube cultures were lysed, blotted and normalized to a ponceau total protein stain. An increase of HYOU1 was detectable in p.Q1662X myotubes but not in the p.Y2704X variant. DNAJC10 was significantly increased in both variants’ myotubes. ANOVA is performed and statistical significance is denoted as following (ns: not significant, * p < 0.05, ** p < 0.01, and *** p < 0.001).


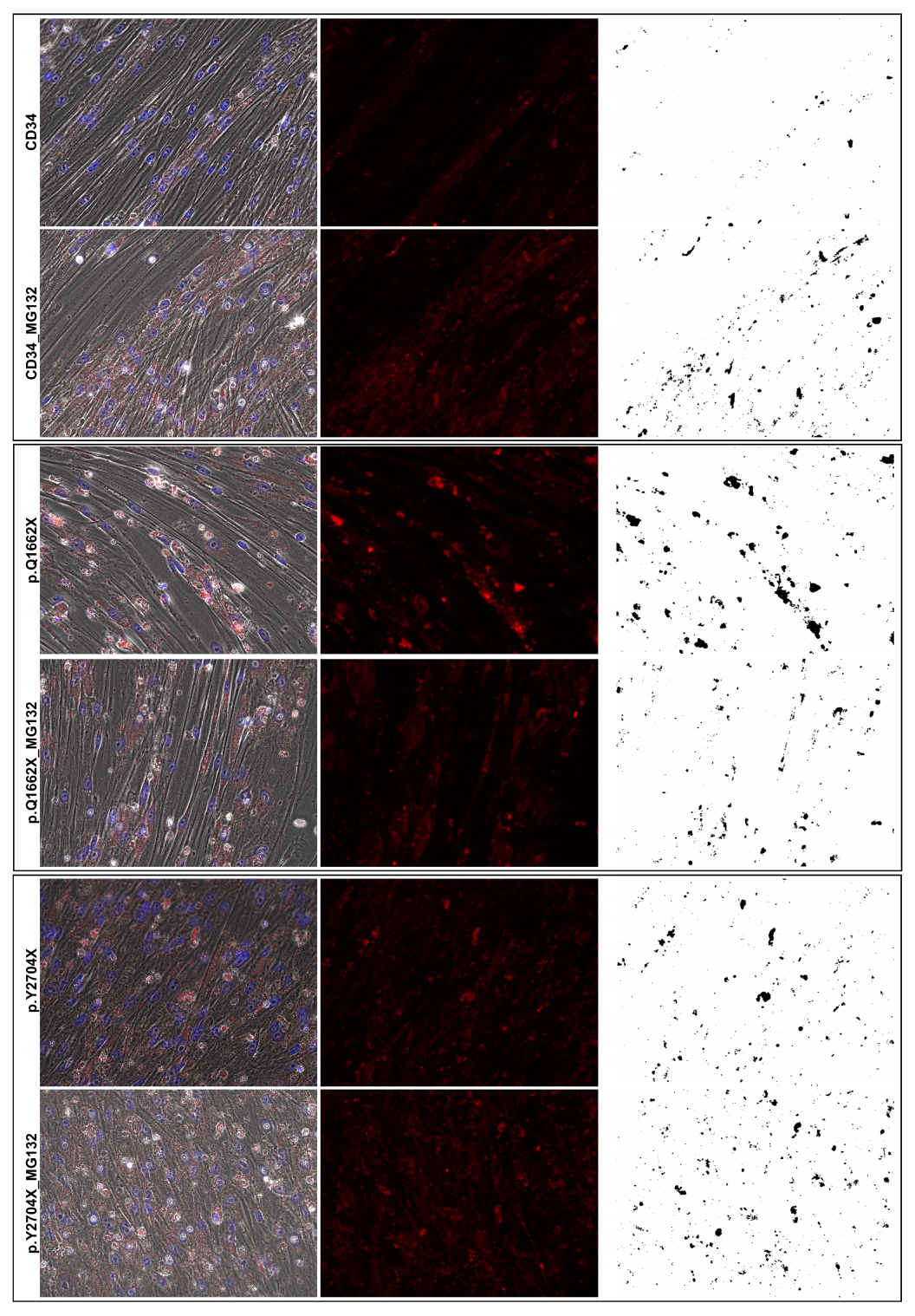


**Supplementary Figure 5. Representative images of the aggresome assay in 2D myotubes.** The left panel shows an overlay of phase-contrast, DAPI (blue) and aggresome staining (red). The middle panel shows the isolated aggresome staining channel. The right panel shows the corresponding binary masks created and used for quantification.
